# A comparative assessment of deep learning and knowledge-based dose prediction models for advanced radiotherapy planning of prostate cancer with focal boosting

**DOI:** 10.1016/j.phro.2026.100977

**Published:** 2026-04-23

**Authors:** Maria A. Piliero, Antonio Angrisani, Davide G. Bosetti, Margherita Casiraghi, Francesco Castronovo, Nicolas Vial, Salvatore Cozzi, Matteo Coppotelli, Klaudia Krzekotowska, Lisa Milan, Francesco Pupillo, Stefano Presilla, Thomas Zilli

**Affiliations:** a Medical Physics, Imaging Institute of Southern Switzerland, EOC, Bellinzona, Switzerland; b Radiation Oncology, Oncology Institute of Southern Switzerland (IOSI), EOC, Bellinzona, Switzerland; c Radiation Oncology, University Hospital of Saint-Etienne, Saint-Priest-en-Jarez, France; d Faculty of Biomedical Sciences, Università della Svizzera Italiana, Lugano, Switzerland; e Faculty of Medicine, University of Geneva, Geneva, Switzerland

**Keywords:** Prostate cancer, Radiotherapy planning, VMAT, Dose prediction, Deep learning

## Abstract

This study compared knowledge-based and deep-learning dose prediction models with clinically approved plans for prostate radiotherapy with focal boosting. The knowledge-based model accurately reproduced clinical plans. Deep-learning predictions showed median variations of 5% (IQR < 5%) for bladder and rectum in high-dose regions and 3.8 Gy higher mean dose (IQR 1.2 Gy) for the femoral heads. However, pudendal artery mean doses were 15 Gy higher (IQR: 10–19 Gy) and urethral V_62.4Gy_ exceeded 50%, above the 2% constraint, likely reflecting their absence in the training dataset. While deep-learning models provide a consistent spatial framework for plan optimization, expert review remains essential.

## Highlights

• Knowledge-based model align with local priorities and clinical treatment plans.
• Deep-learning model focused on target coverage, increasing dose to adjacent organs.
• Deep-learning model showed 5% median volume difference for bladder and rectum.
• Deep-learning predicted 15 Gy higher mean dose for pudendal arteries.
• Careful commissioning and expert review are essential.

## Graphical abstract

**Figure:**
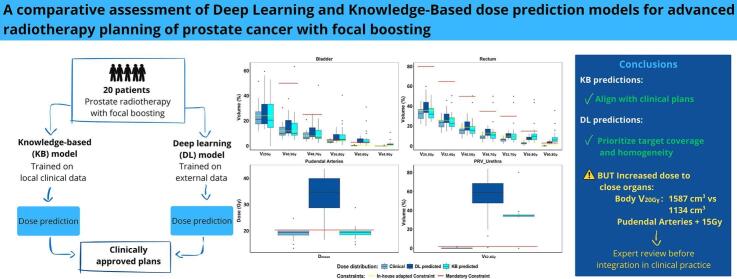

## 1 Introduction

Plan optimization is a crucial step in radiotherapy, ensuring that the distribution of radiation dose is customized to anatomical and tumor characteristics of each patient. Traditionally it is performed manually through an iterative trial-and-error approach. This process requires significant expertise and time investment, with the planner's skill substantially influencing the quality of the final treatment plan [1], [2].

The advancement of new technologies, dose-escalated treatment protocols, and requirements for steep dose gradients for organs of interest sparing, has increased the complexity of radiotherapy planning, making planner expertise increasingly crucial for achieving optimal plan quality. To address these challenges, automated or semi-automated treatment planning tools can help standardize plan quality across radiotherapy centers, while reducing planning time [3], [4], [5], [6]. These tools range from knowledge-based (KB) planning to artificial intelligence approaches using deep-learning (DL) models that can guide the optimization process [7], [8], [9], [10], [11]. This approach provides valuable insights, such as potential dose distributions based on patient-specific anatomic peculiarities that can enhance the planning process, especially in complex cases.

Hypofractionated radiotherapy with focal boosting to the dominant intraprostatic lesion (DIL) is emerging as a new standard in the treatment of localized prostate cancer, providing an effective balance between improved disease control and an acceptable toxicity profile [12], [13]. However, inclusion of a focal boost introduces additional optimization complexity, as it requires steep dose gradients to achieve adequate dose escalation to the DIL while maintaining strict dose constraints for surrounding organs of interest.

In this study, we compared a DL–based dose prediction algorithm, specifically developed to guide prostate cancer radiotherapy planning, with a KB approach in patients with localized prostate cancer treated with moderate hypofractionated radiotherapy and focal boosting to the DIL. The performance of both models was evaluated by comparing predicted dose metrics with those achieved in clinically approved plans, with the aim of assessing whether these predictions could serve as a reliable starting point for plan optimization.

## 2 Material and methods

Data from twenty patients with localized prostate cancer undergoing 20-fraction hypofractionated radiotherapy were analyzed. The study was approved by the local ethical committee (2026–00604, Rif.CE 5111).

Each patient followed a Magnetic Resonance (MR)-Only workflow for radiotherapy treatment planning. MR images were acquired with the patient in supine position using a Philips Ingenia Ambition 1.5 T scanner (Philips Healthcare, Best, The Netherlands) and the Philips' MRCAT software (v.4, Philips Oy, Vantaa, Finland) [14] for synthetic computed tomography (CT) images generation (1.1 mm resolution along the three dimensions).

The DIL was identified and contoured using a combination of Prostate-Specific Membrane Antigen (PSMA) positron emission tomography (PET) imaging co-registered with multiparametric MR imaging, which included diffusion-weighted sequences specific for DIL delineation [13].

The seminal vesicles and the prostate, including the DIL, were planned with different planning target volume (PTV) expansions to create four dose levels according to the DELINEATE [15] protocol. The specific margins used for the PTV expansions were: 3-mm with a 0-mm posterior margin for the PTV_pros_60Gy (prostate); 6-mm with a 3-mm posterior margin for the PTV_SV_57.6 Gy (prostate and base of seminal vesicles); 6-mm isotropic margin for the PTV_SV_48.6 Gy (prostate and entire seminal vesicles), and a 3-mm isotropic margin for the high dose DIL target (PTV_DIL_67Gy).

Doses were delivered in 20 daily fractions via a simultaneous integrated boost (SIB) technique: 48.6 Gy and 57.6 Gy to the distal and proximal seminal vesicles, respectively, 60.0 Gy to the prostate, and 67.0 Gy to the DIL. Organs of interest included the bladder, the rectum, the femoral heads, the penile bulb, the pudendal arteries, and the intraprostatic urethra with a 2-mm isotropic margin as planning risk volume (PRV_urethra) [16], [17]. Specific PTVs coverage and organs of interest dose constraints are detailed in Table S1 in the additional material.

The clinically approved plans, defined as the final treatment plans delivered to the patients after being reviewed and approved by both a medical physicist and a radiation oncologist, were optimized on the Eclipse™ treatment planning system (v. 16.1, Varian Medical Systems, a Siemens Healthineers company, Palo Alto, CA, USA) using the Acuros XB algorithm for dose calculation. The volumetric modulated arc therapy technique (VMAT) with the 6 MV Flattening Filter Free photon beam of a TrueBeam linear accelerator was employed. Each plan included two full arcs, with collimator angles ranging from 315°/45° to 345°/15°. The collimator angles were selected by the planner based on the patient's anatomy.

The optimization of the clinically approved plans was initiated using an in-house developed RapidPlan™ model (Varian Medical Systems, a Siemens Healthineers company, Palo Alto, CA, USA). However, manual adjustments were performed, which included fine-tuning optimization objectives and priorities, as well as adding new objectives as necessary during the iterative optimization process, to ensure that the dose to the organs of interest was kept as low as possible without compromising PTV coverage.

RapidPlan™ is a KB planning tool integrated into the Eclipse™ Treatment Planning System that correlates patient geometry and dose from a library of approved plans to generate initial dose–volume histogram (DVH) prediction and corresponding optimization objectives. The RapidPlan™ model developed in our department was trained on 40 clinically approved plans and included all PTVs and organs of interest, as well as cropped PTV structures to control dose coverage. Lower-dose PTVs were cropped from higher-dose PTVs with an additional 1 mm margin. Auxiliary ring structures were also incorporated to refine dose shaping within the patient’s body. Plan normalization was set to the mean dose of the PTV_DIL_67Gy structure. The model was refined using a closed-loop procedure, where the training set was re-planned using an initial model version to ensure the final model was trained on the most consistent and optimized distributions. The patient plans used for model training were independent of those evaluated in this study.

DVH values for the RapidPlan™ predictions, referred in this work as KB predictions, were extracted from the line objectives generated by the model after their generation and without any manual intervention. Line objectives represent the lowest achievable dose-volume metrics relative to the specific model training.

DL-based predictions were generated using Dose+ (MVision AI, Finland), a cloud-based tool specifically developed for prostate radiotherapy. The workflow involves uploading the patient’s CT images and approved structure set in DICOM format to the cloud service, where the algorithm generates a predicted 3D dose distribution that mimics a VMAT delivery pattern. This algorithm supports various prescription strategies, including SIB. The model is locked and non-interactive, meaning users cannot adjust Target-Organs of Interest trade-offs but can configure dose-scaling preferences (target-based or mean-based). Once the predicted 3D dose distribution is generated, it is exported in DICOM RT Dose format and imported into the treatment planning system for DVH extraction and analysis. It should be emphasized that Dose+ does not suggest or generate a deliverable treatment plan. Instead, it predicts a 3D dose distribution intended to serve as a starting point or initial framework for the subsequent treatment plan optimization process [18].

In this study, the CT images and associated approved structure sets were uploaded into the Dose+ software, where the prescribed dose for each PTV volume was defined. To maintain consistency with the clinical plans, the dose distribution normalization was set to a 67 Gy mean dose to the DIL PTV. The resulting predicted 3D dose distributions were subsequently imported into the Eclipse™ treatment planning system for the extraction of the DVH metrics.

The aim of this study was to evaluate the performance of the KB and DL models by comparing their predicted dose metrics with those achieved in the clinically approved plans, which correspond to the manually optimized plans ultimately delivered to the patients.

Statistical comparison between the clinical plans' dose distribution and the predictions by either the KB or the DL approach was carried out for the following DVH metrics: V_24.3Gy_, V_32.4Gy_, V_40.5Gy_, V_48.7Gy_, V_52.7Gy_, V_56.8Gy_, and V_60.8Gy_ for the rectum; V_25Gy_, V_40.5Gy_, V_48.7Gy_, V_56.8Gy_, V_60.8Gy_, and V_64.9Gy_ for the bladder; V_62.4Gy_ for the PRV of the urethra (generated by a 2 mm isotropic expansion of the urethra); V_40Gy_ and V_48Gy_ for the penile bulb; V_30Gy_ and mean dose for the pudendal arteries; mean dose for the femoral heads; and V_20Gy_ and V_30Gy_ for the body. A paired Wilcoxon test, incorporating Holm's correction for multiple comparisons, was performed with the p-value significance level set at 0.05. The statistical analysis was carried out using the stats package in R, v4.3.3 [19].

## 3 Results

**Fig. 1:**
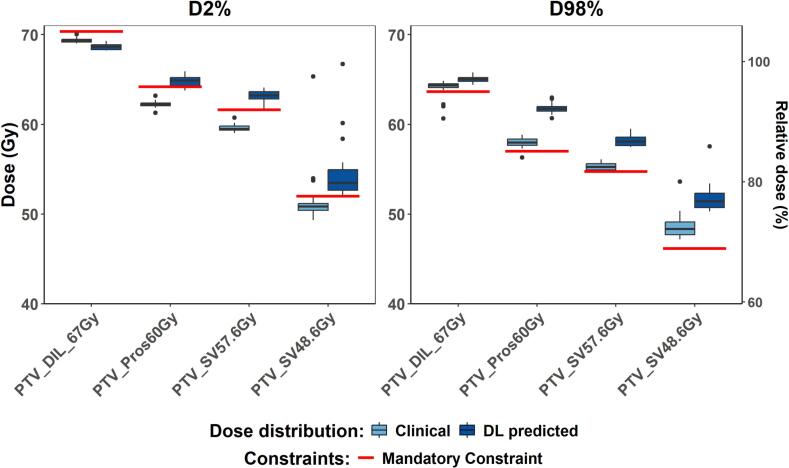
Boxplots of the DVH metric values for the PTVs. The boxplot displays the median value as the central line within the box, with the upper and lower edges of the box representing the 75th and 25th percentiles, respectively. The D_2%_ DVH metric was evaluated on the PTVs cropped volumes, defined as original PTV cropped from the higher dose PTV, with an additional 1 mm margin. The DL-based dose predictions showed greater PTV coverage (higher D_98%_ values) than the clinical plans, but also resulted in higher D_2%_ values that exceeded local requirements. The p-values from the statistical comparisons are summarized in Table S2 of the Additional Material. Abbreviations: DVH, Dose Volume Histogram; PTV, Planning Target Volume.

The KB model generated predictions highly consistent with the clinical dose distributions. In contrast, the DL model predicted dose distributions with higher PTV coverage: median D_98%_ values for the PTV_DIL_67Gy, PTV_Pros60Gy, PTV_SV57.6 Gy, and PTV_SV48.6 Gy were 65 Gy, 61.7 Gy, 58.1 Gy, and 51.4 Gy, respectively, compared to 64.3 Gy, 58 Gy, 55.2 Gy, and 48.3 Gy in the clinical plans (Fig. 1). However, these predictions were also associated with D_2%_ values exceeding the local dose requirements, larger high-dose volumes in organs adjacent to the PTVs and larger low-dose bath throughout the body: median V_20Gy_ and V_30Gy_ within the Body structure were 1587 cm^3^ (IQR: 1202–1740 cm^3^) and 667 cm^3^ (IQR: 512–771 cm^3^), respectively, compared to 1134 cm^3^ (IQR: 844–1342 cm^3^) and 495 cm^3^ (IQR: 365–566 cm^3^) for the clinical plans. Femoral heads mean dose was 3.8 Gy (IQR: 3.3–4.5 Gy) higher.

Below 40 Gy, DL dose predictions remained within the mandatory dose constraints for all organs of interest, except for pudendal arteries, where the median mean dose difference with clinical plans was 15 Gy (IQR: 10–19 Gy) and V_30Gy_ exceeded 50% (versus <10% for clinical plans).

**Fig. 2:**
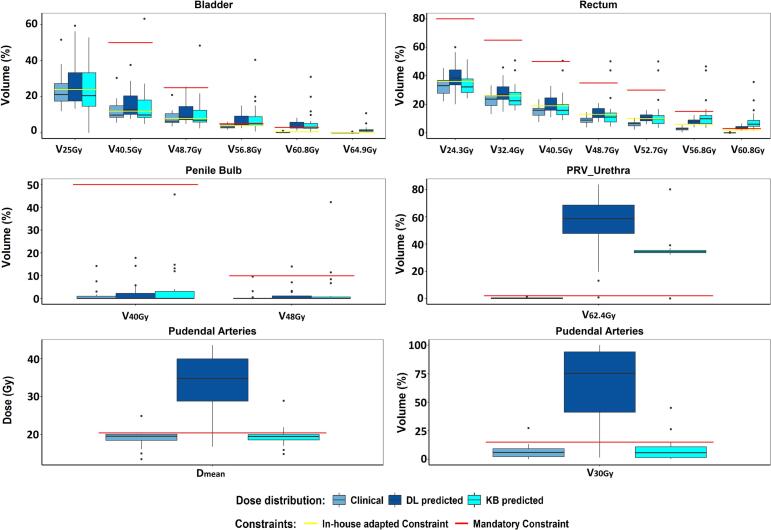
Boxplots of the DVH metric values for the organs of interest. The boxplot displays the median value as the central line within the box, with the upper and lower edges of the box representing the 75th and 25th percentiles, respectively. For the Femoral Heads, the mean dose is reported instead of the V_40Gy_, as the maximum dose values were significantly below 40 Gy. The p-values from the statistical comparisons are summarized in Table S2 of the Additional Material. Abbreviations: DVH, Dose Volume Histogram.

Above 40 Gy, both models predicted DVH values occasionally exceeding mandatory dose constraints. Notably, for the PRV_urethra, the DL model predicted a V_62.4Gy_ exceeding 50%, while the KB model predicted 34.3% (IQR: 33.6%-35.3%) (Fig. 2).

## 4 Discussion

The aim of this work was to evaluate the performance of a knowledge-based approach and a deep-learning-based dose prediction algorithm by comparing their predicted dose–volume histogram metrics with those achieved in clinically approved plans for prostate radiotherapy with focal boosting. The DL model showed modest differences compared to clinical plans for organs of interest, with median variations of 5% (IQR < 5%) for bladder and rectum in high-dose regions and 3.8 Gy (IQR 1.2 Gy) for femoral head mean dose. However, predictions exceeded constraints for the urethra and pudendal arteries, likely due to their absence from the training dataset. The KB model generated predictions highly consistent with the clinical plans, reflecting its training on institution-specific protocols and its routine use in plan initialization. This is consistent with previous studies demonstrating the robustness of knowledge-based approaches in prostate radiotherapy, including SIB techniques [3], [20], [21], [22].

Comparative studies between KBP and DL approaches have reported comparable or improved performance of DL-based models under consistent conditions [23], [24]. The differences observed in this study are likely explained by differences in model training. The KB model was trained on institution-specific treatment plans, whereas the DL model was trained on an external multi-institutional dataset. Consistent with previous reports, DL performance depends on the characteristics of the training dataset and model design [23], [24], [25], [26]. Nevertheless, the relatively small and consistent differences observed for the organs included in the training dataset suggest that the DL model provides a reliable dose framework for standard organs of interest.

These findings highlight the importance of understanding the scope of a dose prediction model and ensuring expert review, particularly for structures not represented in the training data.

In conclusion, the in-house KB model more closely reflected institution-specific planning strategies, whereas the DL model provided consistent and reproducible predictions for standard organs of interest. The limitations found in the DL predictions can be overruled by rescaling or calibrating the planning objectives during the constraint conversion process to meet local institutional requirements. By applying systematic adjustments based on observed performance “deltas,” plan optimization can be personalized to ensure adherence to local sparing protocols while still benefiting from the model’s spatial dose guidance. DL-based dose prediction may therefore serve as a useful starting point for treatment planning, provided that planning objectives are adapted to meet local clinical requirements [8], [10].

## Declaration of generative AI and AI-assisted technologies in the manuscript preparation process

During the preparation of this work the author(s) used NotebookLM in order to refine the clarity of the manuscript. After using this tool, the author(s) reviewed and edited the content as needed and take(s) full responsibility for the content of the published article.

## CRediT authorship contribution statement

**Maria A. Piliero:** Writing – review & editing, Writing – original draft, Visualization, Resources, Methodology, Investigation, Formal analysis, Conceptualization. **Antonio Angrisani:** Writing – review & editing, Resources. **Davide G. Bosetti:** Writing – review & editing, Resources. **Margherita Casiraghi:** Writing – review & editing, Resources. **Francesco Castronovo:** Writing – review & editing, Resources. **Nicolas Vial:** Writing – review & editing, Resources. **Salvatore Cozzi:** Writing – review & editing, Resources. **Matteo Coppotelli:** Writing – review & editing, Resources. **Klaudia Krzekotowska:** Writing – review & editing, Resources. **Lisa Milan:** Writing – review & editing, Resources. **Francesco Pupillo:** Writing – review & editing, Resources. **Stefano Presilla:** Writing – review & editing, Resources. **Thomas Zilli:** Writing – review & editing, Resources, Conceptualization.

## Declaration of competing interest

The authors declare the following financial interests/personal relationships which may be considered as potential competing interests: The Ente Ospedaliero Cantonale (EOC) and the authors affiliated with it did not receive any funding for the conduct of this work, nor will they receive any compensation from MVision AI related to the commercialization of the product.
