# Supplementary material for: A comparative assessment of deep learning and knowledge-based dose prediction models for advanced radiotherapy planning of prostate cancer with focal boosting
Source: Phys Imaging Radiat Oncol. 2026 Apr 23;39:100977. doi: 10.1016/j.phro.2026.100977 (PMC13137215; doi:10.1016/j.phro.2026.100977)
Supplement: Supplementary Data 1 [file mmc1.pdf]

## Supplementary material

**Table S1**: *DVH constraints for target volumes and organs-at-risk for plan quality evaluation. (*) The $D_{2\%}$ was evaluated on the PTVs cropped volume, defined as original PTV cropped from the higher dose PTV, with an additional 1 mm margin. (+) The constraint applies to the planning risk volume (PRV) generated by a 2 mm isotropic expansion of the urethra.*

*Abbreviations: DVH, Dose Volume Histogram; PTV, Planning Target Volume; DIL, Dominant Intraprostatic Lesion; SV, Seminal Vesicles; PRV, Planning Risk Volume.*

| Structure | DVH Metric | In-house adapted Constraint | Mandatory Constraint |
|---|---|---|---|
| **PTV_DIL_67Gy** | $D_{98\%}$ | ≥ 95% | |
| | $D_{2\%}$ | ≤105% | |
| **PTV_pros60Gy*, PTV_SV57.6Gy*, PTV_SV48.6Gy*** | $D_{98\%}$ | ≥ 95% | |
| | $D_{2\%}$ | ≤107% | |
| **Rectum** | $V_{24.3\ Gy}$ | <36% | <80% |
| | $V_{32.4\ Gy}$ | <26% | <65% |
| | $V_{40.5\ Gy}$ | <19% | <50% |
| | $V_{48.7\ Gy}$ | <13% | <35% |
| | $V_{52.7\ Gy}$ | <10% | <30% |
| | $V_{56.8\ Gy}$ | <6% | <15% |
| | $V_{60.8\ Gy}$ | <2% | <3% |
| **Bladder** | $V_{25\ Gy}$ | | <24% |
| | $V_{40.5\ Gy}$ | <12% | <54% |
| | $V_{48.7\ Gy}$ | <8% | <25% |
| | $V_{56.8\ Gy}$ | <4% | <5% |
| | $V_{60.8\ Gy}$ | <0.6% | <3% |
| | $V_{64.9\ Gy}$ | | <0.2% |
| **PRV_Urethra+** | $V_{62.4\ Gy}$ | | <2% |
| **Penile Bulb** | $V_{40\ Gy}$ | | <50% |
| | $V_{48\ Gy}$ | | <10% |
| **Pudendal Arteries** | $V_{30\ Gy}$ | <15% | |
| | $D_{mean}$ | < 20.4 Gy | |
| **Femoral Head Left/Right** | $V_{40\ Gy}$ | | <5% |

**Table S2:** *P-values resulting from the statistical comparison between the clinical plans' dose distribution and the predictions by either Rapid Plan™ or the DL approach. A paired Wilcoxon test, incorporating Holm's correction for multiple comparisons, was carried out.*

*Abbreviations: DIL, Dominant Intraprostatic Lesion; SV, Seminal Vesicles; PRV, Planning Risk Volume.*

| Structure | | Clinical VS DL Prediction | Clinical VS RP Prediction | DL Prediction VS RP Prediction |
|---|---|---|---|---|
| **Rectum** | $V_{24.3\,Gy}$ | 0.01 | 0.4 | 0.2 |
| | $V_{32.4\,Gy}$ | 0.001 | 0.7 | 0.1 |
| | $V_{40.5\,Gy}$ | $1*e^{-5}$ | 0.7 | 0.1 |
| | $V_{48.7\,Gy}$ | $1*e^{-5}$ | 0.002 | 0.2 |
| | $V_{52.7\,Gy}$ | $1*e^{-5}$ | $5*e^{-4}$ | 0.3 |
| | $V_{56.8\,Gy}$ | $1*e^{-5}$ | $1*e^{-5}$ | 0.1 |
| | $V_{60.8\,Gy}$ | $1*e^{-5}$ | $1*e^{-5}$ | 0.001 |
| **Bladder** | $V_{25\,Gy}$ | $4*e^{-4}$ | 0.8 | 0.9 |
| | $V_{40.5\,Gy}$ | $1*e^{-5}$ | 0.9 | 0.8 |
| | $V_{48.7\,Gy}$ | $1*e^{-5}$ | 0.9 | 0.9 |
| | $V_{56.8\,Gy}$ | $1*e^{-5}$ | $8*e^{-4}$ | 1 |
| | $V_{60.8\,Gy}$ | $3*e^{-4}$ | $1*e^{-5}$ | 1 |
| | $V_{64.9\,Gy}$ | 0.02 | $1*e^{-5}$ | $1*e^{-5}$ |
| **PRV_Urethra+** | $V_{62.4\,Gy}$ | $2*e^{-6}$ | $1*e^{-4}$ | 0.008 |
| **Penile Bulb** | $V_{40\,Gy}$ | 0.04 | 0.4 | 1 |
| | $V_{48\,Gy}$ | 0.04 | 0.4 | 1 |
| **Pudendal Arteries** | $V_{30\,Gy}$ | $2*e^{-6}$ | 0.4 | $1*e^{-5}$ |
| | $D_{mean}$ | $2*e^{-6}$ | 0.4 | $6*e^{-6}$ |
| **Femoral Head Left** | $D_{mean}$ | $2*e^{-6}$ | 0.3 | $1*e^{-4}$ |
| **Femoral Head Right** | $D_{mean}$ | $1*e^{-4}$ | 0.2 | $1*e^{-6}$ |
| **Body** | $V_{20\,Gy}$ | 0.001 | -- | -- |
| | $V_{30\,Gy}$ | 0.001 | -- | -- |
